# Usefulness of preoperative venography in patients with cardiac implantable electronic devices submitted to lead replacement or device upgrade procedures

**DOI:** 10.5935/abc.20180164

**Published:** 2018-11

**Authors:** Caio Marcos de Moraes Albertini, Katia Regina da Silva, Joaquim Maurício da Motta Leal Filho, Elizabeth Sartori Crevelari, Martino Martinelli Filho, Francisco Cesar Carnevale, Roberto Costa

**Affiliations:** 1 Instituto do Coração (InCor) - Faculdade de Medicina da Universidade de São Paulo, São Paulo, SP - Brazil; 2 Hospital das Clinicas da Faculdade de Medicina da Universidade de São Paulo, São Paulo, SP - Brazil

**Keywords:** Pacemaker, implantable defibrillators, phlebography, venous stenosis, extraction of leads, risk factors

## Abstract

**Background:**

Venous obstructions are common in patients with transvenous cardiac implantable electronic devices, but they rarely cause immediate clinical problems. The main consequence of these lesions is the difficulty in obtaining venous access for additional leads implantation.

**Objectives:**

We aimed to assess the prevalence and predictor factors of venous lesions in patients referred to lead reoperations, and to define the role of preoperative venography in the planning of these procedures.

**Methods:**

From April 2013 to July 2016, contrast venography was performed in 100 patients referred to device upgrade, revision and lead extraction. Venous lesions were classified as non-significant (< 50%), moderate stenosis (51-70%), severe stenosis (71-99%) or occlusion (100%). Collateral circulation was classified as absent, discrete, moderate or accentuated. The surgical strategy was defined according to the result of the preoperative venography. Univariate analysis was used to investigate predictor factors related to the occurrence of these lesions, with 5% of significance level.

**Results:**

Moderate venous stenosis was observed in 23%, severe in 13% and occlusions in 11%. There were no significant differences in relation to the device side or the venous segment. The usefulness of the preoperative venography to define the operative tactic was proven, and in 99% of the cases, the established surgical strategy could be performed according to plan.

**Conclusions:**

The prevalence of venous obstruction is high in CIED recipients referred to reoperations. Venography is highly indicated as a preoperative examination for allowing the adequate surgical planning of procedures involving previous transvenous leads.

## Introduction

Venous obstructions frequently occur in patients with transvenous cardiac implantable electronic devices (CIED), with an estimated 14 to 64% prevalence.^1^^-^^11^ Those lesions are mostly asymptomatic, although visible collateral circulation in the thoracic region is usually found. Although deep venous thrombosis, pulmonary thromboembolism, or superior vena cava syndrome were found in 1.6 to 12% of the cases, the difficulty in gaining access to implant new additional leads or other types of transvenous devices has been the main consequence of those lesions.^12^^-^^16^

Recent studies have shown an increase in the number of reoperations in which it is necessary to handle the intravascular territory with leads previously implanted.^17^^-^^23^ The increase in this type of procedure is due to three main factors: (1) patients’ increasing longevity, which is directly related to the longer period of time leads remain in the territory and, consequently, to a greater chance of dysfunction of the stimulation system’s components; (2) an increase in comorbidities leading to an increase in the occurrence of infectious complications, whose treatment necessarily requires the complete CIED removal^17^^-^^23^ and (3) an increasing prevalence heart failure and, consequently, of the need to upgrade from the conventional pacemaker to more advanced modes, such as implantable cardioverter-defibrillator (ICD), or cardiac resynchronization therapy (CRT), which require the implantation of additional leads.^24^^-^^27^

Digital subtraction venography provides excellent characterization of the venous anatomy and has been deemed the gold standard for studying venous lesions in CIED patients.^11^^,^^28^^-^^30^ Although other imaging techniques are used for the same purpose, such as Doppler ultrasonography or contrast recirculation in thoracic computed tomography images, these methods are not as accurate as digital venography to quantify and define where obstructions are located and any collateral circulation developed.^31^^-^^34^

This study is part of a prospective registry, with data derived from medical practice, and its goals are: (1) to identify the prevalence, degree and location of venous lesions in CIED patients with an indication of reoperation; (2) to identify predisposing factors of these venographic changes; and (3) to define the role of digital subtraction venography when intravascular reinterventions are planned in individuals with leads previously implanted.

## Methods

### Study Design and Population

This is a cross-section analysis derived from a cohort where thromboembolic complications are studied in patients submitted to lead revision or upgraded procedures. This study was conducted in a high-complexity cardiology hospital and it was approved by that hospital’s Committee of Ethics in Research. All subjects signed a free and informed consent form.

From April 2013 to July 2016, patients who met the following criteria were consecutively included: (1) having CIED implanted at the territory of the superior vena cava for more than six months; (2) being between 18 and 90 years of age; (3) having an indication for lead revision or upgrade procedures. The following candidates were not included: (1) individuals with creatinine > 1.5 mg/dL due to the risk of renal damage from iodinated contrast; (2) candidates that had known allergy to iodinated contrast media; and (3) those who declined to participate in the study.

Considering the high rates of venous lesions in these patients, a convenience sample of 100 patients was defined to detect the outcomes studied.

### Study Outcomes

The outcomes of the study included: (1) venographic findings of significant venous obstructions and collateral circulation, and (2) usefulness of the preoperative venographic findings when planning and performing the surgical procedure.

### Study Workflow

Patients with an indication of reoperation for implantation of additional leads, replacement or removal of previously-implanted transvenous leads, and who met the eligibility to the study were submitted to preoperative evaluation comprising patient background assessment, clinical evaluation and evaluation of imaging exams.

Thorax radiography was conducted to help determining the position of the leads in use or abandoned.

The venous system was evaluated using digital subtraction venography through images acquired with an Allura DSA unit or Allura Xper FD20 (Philips, The Netherlands) to bilaterally assess the axillary, cephalic, subclavian, innominate (or brachiocephalic trunk) veins, and superior vena cava. Continuous infusion of low-osmolality nonionic iodinated contrast media (Visipaque-Iodixanol, 320 [652 mg/mL Iodixanol], GE, Healthcare, Europe) was performed using a MEDRAD injection pump with controlled volume (100 mL to 120 mL) and infusion speed (10 mL/s at 600 psi pressure). All exams were simultaneously evaluated by two specialists: a Vascular Interventional Radiologist and a Cardiac Pacing Specialist.

The images obtained were classified according to the presence or absence of venous lesions and of collateral circulation. Venous lesions were classified according to their stenosis level: without significant alteration (< 50%), moderate stenosis (51-70%), severe stenosis (71-99%), and occlusion (100%).

### Surgical Procedures

Surgical procedures were performed according to the hospital’s usual routines, always under the supervision of an anesthesiologist. Operations were grouped in three main types: (1) Implanting new leads without further removal (due to dysfunction of a previously implanted lead, or upgrade procedures); (2) Replacing leads with the removal of previously implanted leads; or (3) Isolated lead extraction.

Operations were planned according to the radiological function of the venous territory obtained through venography: (1) In cases where the venous pattern was deemed without significant lesions or with moderate lesions, no special care was taken to implant new leads and, similarly, the decision of removing a deactivated lead was made at the surgical team’s discretion. (2) In cases with stenosis deemed severe or occlusions, surgical planning considered: a) careful evaluation of the venography to check the possibility of using the ipsilateral internal jugular vein; b) preparing the patient for transvenous lead extraction to provide access for the new lead when using the ipsilateral internal jugular was not possible; c) reserving material for attempts to go beyond a lesion and perform venous dilation.

The decision whether to remove or abandon in situ the previously abandoned leads or the ones that would be deactivated in the current surgical procedure was made considering the following criteria: (1) patient’s age and life expectancy; (2) number of leads remaining in the superior vena cava at the end of the surgical procedure performed in this study; (3) risk of worsening the lesions observed in the venography.

Although the criteria for defining an access to deactivated leads and whether to remove or abandon them were previously discussed with the surgical team involved in the study, the final decision on both topics was to be made by the team itself during the procedure due to the intraoperative findings and technical resources available.

### Agreement between Planned and Actually Performed Procedure

To assess the agreement between the procedure planned according with the venography findings and the procedure actually performed, three conditions were considered: (1) possibility of access to the heart by the subclavian vein without any special strategies; (2) possibility of access to the heart by the ipsilateral internal jugular vein when there was a severe lesion or subclavian vein occlusion; (3) whether lead extraction or other unconventional technique was required to gain access in cases of critical lesion affecting the subclavian vein, internal jugular vein and venous brachiocephalic trunk.

### Care Provided for Study Subjects

The risks associated with the present study were related to the use of iodinated contrast media. Special care was taken to reduce the risk of renal damage following digital subtraction venography, although adverse reactions related to the use of non-ionic iodinated contrast agents are rare. Diabetic patients receiving oral hypoglycemic metformin hydrochloride were instructed to discontinue the use of that drug for 48 hours before the test and resume use 48 hours after the test. The cases of allergic reactions to iodinated contrast during or after the exams were treated according to the institution’s protocol for allergic reactions to contrast.

### Electronic Data Collection and Management

The demographic, clinical and surgical data obtained were stored at the database developed in the REDCap system (*Research Electronic Data Capture*)^35^ hosted at the hospital’s server.

### Variables Studied and Statistical Analysis

The following data were analyzed as independent variables for the risk of occurrence of the outcomes studied: demographic data, preoperative clinical data at baseline, type of CIED, and type of procedure performed.

The data recorded in the database (REDCap) were exported in the format of Excel worksheets (*Microsoft Excel*) and analyzed using SAS software (*Statistical Analysis System*).

Initially all variables were analyzed descriptively. The quantitative variables were analyzed by considering the minimal and maximum values, means, standard deviation and median. The qualitative variables were analyzed by calculating the absolute and relative frequencies. We compared means using Student t-test, and tested homogeneity among the variable proportions using chi-square test. The significance level chosen for statistical tests was 5%.

The outcomes of the study were described according to absolute and relative frequencies. The calculation of *Odds Ratio* (OR) and its confidence intervals at 95% were used as an effect measure between exposure variables and outcome development.

## Results

**Figure 1:**
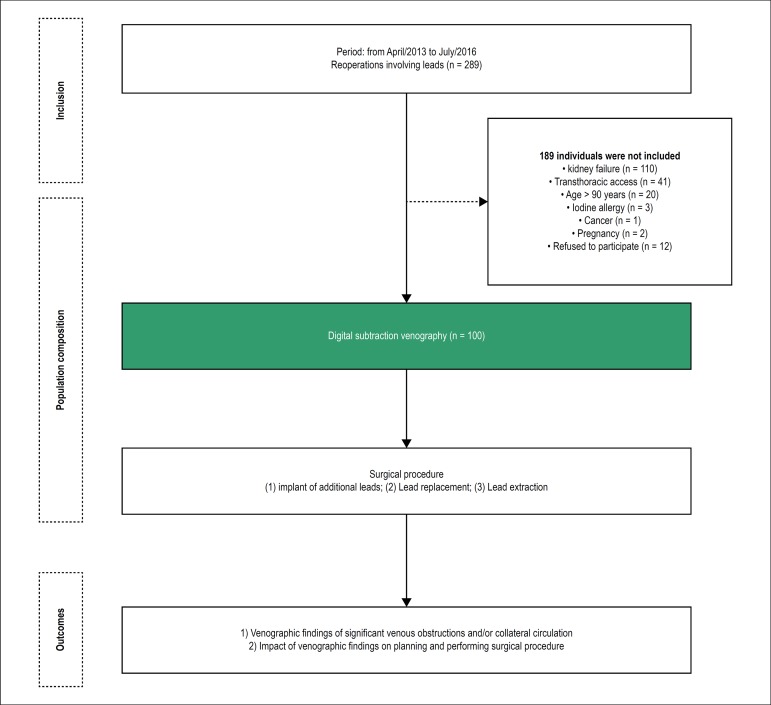
Composition of the population studied and Study phases.

Of 289 patients with an indication of reoperation involving the handling of leads, 100 were included in this study. (Figure 1)

The population was balanced with regard to gender, had a predominance of Caucasian individuals (82%) and a mean age of 58.5 ± 15.1 years, with median 60. Most individuals studied were oligosymptomatic for heart failure (77%), with a left ventricular ejection fraction of 53.4 ± 15.5, 39% of which had no structural cardiac disease identified. Only 20% of cases did not have any comorbidity. One third of this population was using antiplatelet agents, while anticoagulants were used by 12% of the patients (Table 1).

**Table 1:** Demographic and clinical characteristics of the study subjects

Demographic and Clinical characteristics at baseline	
Male, n (%)	48%
Age (years), means ± DP	58.5 ± 15.1
Caucasian, n (%)	82%
Body mass index, means ± DP	25.7 ± 3.2
**Functional class (NYHA), n (%)**	
I	40%
II	37%
III	23%
**Structural heart disease, n (%)**	
None	39%
Chagas disease	23%
Ischemic heart disease	8%
Non-ischemic heart disease	24%
Other	6%
**Associated comorbities**	
None	20%
Systemic arterial hypertension	62%
Diabetes	17%
Dyslipidemia	33%
Coronary arterial disease	9%
Valvopathy	7%
Smoker (current)	1%
Smoker (previously)	9%
**Medicines being used, n (%)**	
Antiplatelet agents	33%
Oral anticoagulants	12%
Statins	39%
Left ventricular ejection (%) means ± DP	53.4 ± 15.5

SD: Standard deviation; NYHA: New York Heart Association.

There was a balance in the number of cases with devices implanted on the right side (48%) and those on the left side (52%). Marking differences were observed, however, concerning time since implantation, with an average 14.3 ± 6.1 years for the right side, and 8.0 ± 7.9 years for the left side; as to the type of device, there were more conventional pacemakers on the right, while the four device types were more evenly distributed for the left side. (Table 2)

**Table 2:** Characteristics of the cardiac device being used at the time of inclusion in the study according to the side of the implant

Characteristics of the previous CIED	Right side (n = 48)	Left side (n = 52)	p
**Type of CIED, n (%)**			
Conventional pacemaker	45	31	
Conventional ICD	1	18	< 0.001 ^(1)^
CRT	1	1	
CRT-D	1	2	
**Total number of transvenous leads, n (%)**			
One	10	12	
Two	33	37	0.306 ^(1)^
Three	4	3	
Four	1	-	
**Dwelling time of transvenous leads, years**			
Means ± SD	14.3 ± 6.1	8.0 ± 7.9	0.075 ^(2)^
Variation	5 - 37	1 - 32	

CIED: cardiac implantable electronic device; ICD: implantable cardioverter-defibrillator; CRT: cardiac resynchronization therapy; CRT-D: cardiac resynchronization therapy associated with implantable cardioverter-defibrillator.

(1) Chi-square test

(2) Student t-test

### Results of Digital Subtraction Venography

**Figure 2:**
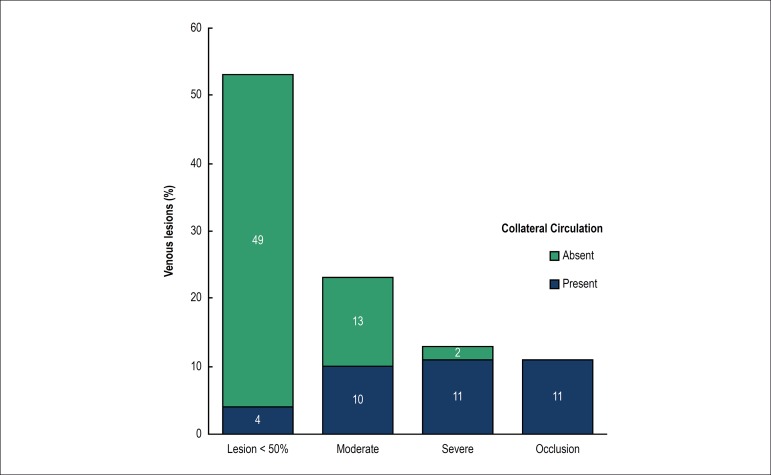
Distribution of the four types of venous lesions and their associations with the presence of collateral circulation.

**Figure 3:**
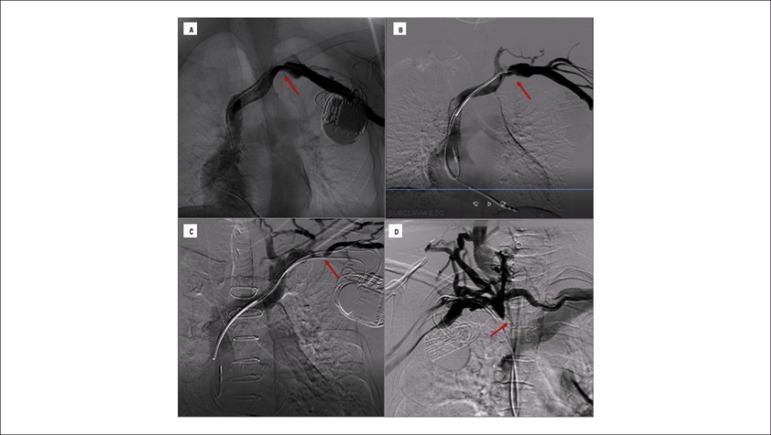
Classification of venous lesions and collateral circulation. Examples of the four types of lesion according to the classification adopted in the study. Figure 3A: non-significant lesions characterized with obstruction of less than 50% of the blood vessel lumen and absence of collateral circulation; Figure 3B: moderate lesion in 51% to 70% of the vessel, with discrete collateral circulation; Figure 3C: severe lesion compromising 71% to 99% of the vessel with moderate collateral circulation; Figure 3D: venous occlusion with accentuated collateral circulation.

Analyses of the venographies showed that 47 patients had significant venous lesions and that in 36 out of those there was venous collateral circulation. Moderate venous obstructions were observed in 23 exams, severe in 13, and occlusions in 11. Of the 53 patients without significant obstructions (< 50% of blood vessel lumen), only 4 had collateral circulation. On the other hand, out of the 24 individuals with venous lesion deemed severe or with venous occlusion, just 2 did not present collateral circulation in their venography. Therefore, finding collateral circulation in venography was observed to be a strong marker of the presence of venous lesion, increasing 4.9 times the prevalence rate (CI 95% 3.05 - 8.10; p < 0.0001) of those lesions (Figures 2 and 3).

Despite the differences of time since implantation and types of devices implanted, there was balance between the findings of venous lesions (p = 0.865) and of collateral circulation (p = 0.715) in patients with devices implanted on the right and left sides. Regardless of the side the CIED had been implanted, subclavian veins and the transition from subclavian veins to the brachiocephalic trunk were the regions that presented the highest number of significant lesions (Table 3). No significant lesions were identified in the superior vena cava.

**Table 3:** Distribution of venographic findings according to the CIED side and the anatomical location of the lesion

Venographic findings	Right side (n = 48)	Left side (n = 52)
**Normal exam / discrete lesions (< 50% of vessel lumen)**		
Subclavian vein	37	43
Transition from subclavian vein to innominate vein	46	44
Innominate vein	42	46
Joint of innominate vein and superior vena cava	33	46
**Moderate stenosis**		
Subclavian vein	4	5
Transition from subclavian vein to innominate vein	-	4
Innominate vein	1	2
Joint of innominate vein and superior vena cava	8	2
**Severe stenosis**		
Subclavian vein	3	2
Transition from subclavian vein to innominate vein	2	3
Innominate vein	1	1
Joint of innominate vein and superior vena cava	3	3
**Venous occlusion**		
Subclavian vein	4	2
Transition from subclavian vein to innominate vein	-	1
Innominate vein	4	3
Joint of innominate vein and superior vena cava	4	1
**Collateral circulation**		
Absent	19	29
Discrete	13	7
Moderate	5	8
Strong	11	8

### Indication of surgical procedure

The main reason to perform a surgical procedure was lead dysfunction, in 71 patients. Upgrade procedures was the cause of reoperation in 25 cases. Only for 4 patients the operation was caused solely by a need of lead removal (Table 4).

**Table 4:** Characteristics of surgical procedures performed in the study

Characteristics of Surgical Procedures	n = 100
**Procedure performed, (%)**	
Implant of additional lead without removing previously implanted lead	48
Implant of additional lead with removal of previously implanted lead	48
Only lead removal	4
**Total number of transvenous leads at the end of the procedure, (%)**	
None	4
One	6
Two	41
Three	42
Four	7
**CIED side at the end of the procedure, n (%)**	
Right	45
Left	54
Subxiphoid	1

Leads were removed from 52 patients. Transvenous extraction with mechanical or laser sheaths was performed in 36 patients, while leads were removed through simple traction in just 16 cases. At the end of the operation, only 4 patients remained without any transvenous lead implanted, and in most cases (90%), two or three leads remained in the venous territory.

### Usefulness of Venography to Define Surgical Planning

Agreement between the surgical strategy based on the analysis of digital subtraction venography and the surgical procedure actually performed occurred in 99 out of the 100 patients operated. Lack of agreement, which occurred with a single patient, arose from a mistake in classifying the degree of a lesion in the right subclavian vein, which was deemed moderate in the preoperative period, but during the operation was found to be a sub-occlusive lesion (Table 5).

**Table 5:** Agreement between the surgical strategy defined using preoperative venography and the surgical procedure performed

Surgical planning	Cases planned	Cases performed
• Venous stenosis < 50% to moderate stenosis	76	75
Direct access through the cephalic subclavian/cephalic vein		
• Severe stenosis or occlusion, with jugular vein and/or brachiocephalic trunk without obstructive lesions	11	11
Access through internal jugular vein		
• Severe stenosis or occlusion, with jugular vein and/or brachiocephalic trunk with obstructive lesions	13	14
Lead extraction		

In all the cases studied, surgical planning was based on the findings of preoperative venography. Of the 53 patients without significant lesions, there were 28 cases in which we decided to implant new leads without removing the old ones, while in 22 cases the implantation of new leads was combined with removal of old ones in order to avoid overpopulation. There was complete removal of the system in other 3 cases.

On the other hand, of the 23 cases where moderate stenosis had been diagnosed, there were 14 in which there was the implantation of new leads combined with the removal of old ones; only in 9 cases our decision was to implant new leads and maintain the old ones.

In the 24 cases where new leads did not require any removal and severe stenosis or venous occlusion had been diagnosed, the findings in the venography showed that in 13 cases the internal jugular vein and the ipsilateral brachiocephalic trunk of the implant were free from any obstructions. Of those, only in 2, because the patients were young, a transvenous extraction procedure was planned to avoid overpopulation of leads. Of the 11 cases where no extraction was performed, there were 5 in which the internal jugular vein was used as access. In the other 5 cases, it was possible to go beyond the lesion in the subclavian vein with the aid of 0,14” hydrophilic wire guides. Of the 8 cases where the internal jugular veins could not be used as access because there was obstruction in the ipsilateral venous brachiocephalic trunk, in only one case the medical team chose to conduct a new contralateral implantation. In the remainder (7), transvenous extraction was the chosen access.

Leads were removed without implanting new ones in only 4 cases: in 3, to treat an infection related to the device, and in 1 to remove a dysfunctional lead which was causing noise in an ICD. In this last case the venography showed venous occlusion.

### Prognostic Factors of Venographic Alterations

**Figure 4:**
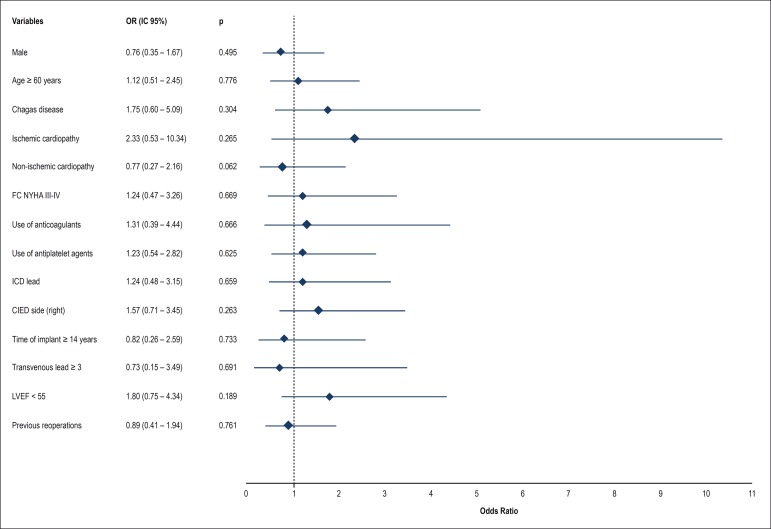
Risk factors for the occurrence of significant venous lesions (> 50% of obstruction of blood vessel lumen) and/or presence of collateral circulation.

Despite the high rate of venographic outcomes in the patients studied, it was not possible to identify prognostic factors for the occurrence of venographic alterations. The following variables were tested as probable prognostic factors: gender, age at the time of the venographic study, cardiopathy at baseline, functional class for heart failure, use of oral anticoagulants and antiplatelet agents, having an ICD lead, CIED implantation side, time since CIED implantation, number of leads implanted, left ventricular ejection, and previous procedures of reoperation (Figure 4).

## Discussion

Venous obstructions seldom cause immediate clinical problems. However, when new leads have to be implanted, the presence of those lesions can make the procedure impossible with conventional techniques. Thus, digital subtraction venography has been mostly used because it allows identifying precisely how serious venous lesions are, as well as their location, thus allowing the planning of proper surgical strategy.^11^^,^^28^^-^^30^

The high prevalence of individuals with lesions deemed significant in this study was compatible with other experiences reported in the literature.^1^^-^^11^ Regardless of lesion seriousness, their distribution was balanced among the subclavian veins, the venous brachiocephalic trunk or the transitional areas of those veins.

Despite the particularities existing among the anatomy of the veins draining the left side and the right side of the thorax, the venographic study did not identify significant differences in the frequency of those findings, in how serious the stenosis was, or in the location of the lesions between the two sides. However, there were differences in the average time leads had remained implanted, i.e., longer for patients who had the device implanted on the right side, which may have increased the rate of occurrences of lesions in the right territory. On the other hand, despite the balance between the numbers of leads implanted, the number of defibrillator leads, which is deemed a risk factor for venous lesions, was significantly higher in the cases where the CIED had been implanted on the left side.^1^^-^^4^^-^^8^

The strong association between the presence of collateral circulation and severe or occlusive venous lesions, which was observed in this study, is quite useful to interpret venographies. Therefore, we can say that whenever there is collateral circulation, lesions difficult to be defined have to be carefully looked for. In this respect, we suggest maintaining dynamic venography images, which allow following the iodinated contrast path. Often enough, when the contrast passes exclusively through the collateral circulation, it fully fills up the blood vessel lumen soon after the critical lesion, which prevents it from being detected in still images.

The high rate of patients with severe or occlusive lesions observed in this study, which agrees with the data in the literature, evidenced the importance of venography for surgical planning. In cases where significant venous lesions could not be identified, the surgical team were able to plan a procedure in which deactivated leads should (or should not) be extracted by considering solely factors such as patient age or the number of leads that would remain in the venous territory. On the other hand, in patients where moderate lesions were observed, the medical team could plan which leads should be extracted in order to avoid an overpopulation of leads that could worsen obstructions. And, finally, in the cases where severe or occlusive venous lesions were observed, the knowledge of the venous anatomy was of essence to plan the surgery, since it raises the possibility of using the ipsilateral jugular vein or the need of extracting leads to gain proper access.

Since causes are multifactorial, the literature is controversial as to defining predictive factors of thromboembolic complications in CIED patients.^2^^-^^11^^-^^36^^-^^37^ In this respect, the absence of risk factors for venous lesions found in this study sample confirms the importance of preoperative venography in patients requiring lead reoperations, since it was not possible to identify any subgroup of individuals less subject to venous obstructions.

### Study Limitations

Although this study is part of a prospective registry derived from medical practice, due to the non-inclusion criteria used, our conclusions cannot be extended to children, to individuals over 90 years of age and to those with renal dysfunction with serum creatinine over 1,5 mg/dL.

As to the rate of venous alterations found and their predisposing factors, this analysis has the same limitations as other cross-sectional studies, as they were assessed at a particular time.

## Conclusions

The high prevalence of severe obstructions or venous occlusions in CIED patients makes a transvenous implant of new leads difficult in a considerable number of patients. Sometimes, using non-conventional techniques, such as the extraction of leads to achieve access, can be mandatory. The lack of predisposing factors and the absence of clinical signs of venous obstruction, which occurs in most patients with severe or occlusive lesions, can hinder the planning of a surgery. Thus, digital subtraction venography is quite useful to define a surgical strategy in operations for lead revision or upgrade procedures. The finding of collateral veins in this exam has a high predictive value for diagnosing severe and occlusive lesions.
